# Increasing digital mental health reach and uptake via youth partnerships

**DOI:** 10.1038/s44184-023-00030-1

**Published:** 2023-06-27

**Authors:** Colleen Stiles-Shields, Giovanni Ramos, Adrian Ortega, Alexandra M. Psihogios

**Affiliations:** 1 Institute for Juvenile Research, Department of Psychiatry, College of Medicine, University of Illinois, Chicago, Chicago, IL USA; 2 Center for Health Equity using Machine Learning & Artificial Intelligence, College of Medicine, University of Illinois, Chicago, Chicago, IL USA; 3 Montefiore Medical Center, University of California, Los Angeles, Los Angeles, CA USA; 4 Clinical Child Psychology Program, University of Kansas, Lawrence, KS USA; 5 Department of Medical Social Sciences, Northwestern University, Chicago, IL USA

**Keywords:** Health care, Social sciences

## Abstract

Youth in the United States are facing an unprecedented mental health crisis. Yet, brick-and-mortar mental healthcare, such as face-to-face therapy, is overwhelmingly inaccessible to youth despite research advances in youth mental health. Digital Mental Health tools (DMH), the use of technologies to deliver mental health assessments and interventions, may help to increase mental healthcare accessibility. However, for a variety of reasons, evidence-based DMH have not been successful in reaching youth in real-world settings, particularly those who are most encumbered with access barriers to mental healthcare. This Comment therefore focuses on increasing DMH reach and uptake by young people, particularly among minoritized youth, by engaging in community-based youth partnerships. This idea recognizes and grows from decades’ worth of community-based participatory research and youth partnerships successfully conducted by other disciplines (e.g., social work, public health, urban planning, education). Increasing uptake and engagement is an issue that is unlikely to be solved by adult-driven theory and design. As such, we emphasize the necessity of reframing youth input into DMH design and deployment from one-time participants to integral community-based partners. Indeed, recognizing and valuing their expertise to equitably address DMH implementation challenges, youth should help to pose the very questions that they will help to answer throughout the design and implementation planning for DMH moving forward.

## Introduction

Major institutions are drawing attention to the unprecedented youth mental health crisis in the United States (U.S.)^1^. Responsively, the U.S. Preventive Services Task Force now recommends that all youth over the age of seven be screened for anxiety and those over 12 be screened for depression at primary care appointments^2^. However, increased awareness and screening might not ameliorate a perennial injustice for our nation’s youth: inaccessible mental healthcare. Practical (e.g., time), emotional (e.g., symptom severity), and systemic (e.g., discrimination) barriers consistently block youth from mental healthcare^3^. Recognizing decades’ worth of successful community-based participatory research (CBPR) in other contexts^4^, this Comment centers on increasing access to mental healthcare for youth via digital mental health tools (DMH)–the use of technologies like smartphones, websites, and apps to deliver mental health assessments and interventions–by addressing both DMH reach and uptake via youth partnerships. Specifically, youth over the age of ten^5^ must be involved in the design and evaluation of DMH to increase the likelihood that they engage with these technologies (uptake) and that these tools make it to youth in the first place (reach).

A core criticism of psychological interventions is that they have been developed using “efficacy first” approaches, implemented under the “ideal” conditions^6^. As a result, evidence-based interventions often fail to match the needs of patients or overcome the constraints of clinical care in community settings. Indeed, brick-and-mortar mental healthcare is overwhelmingly inaccessible to youth. Even with the expansion of insurance coverage for telehealth during the COVID-19 pandemic, mental health service use among youth remained somewhat similar or even worsened^7^. Systemic barriers that contribute to healthcare disparities, such as discriminatory practices, cost, travel, and time, also occur in mental healthcare–and are further compounded by issues such as stigma and lack of problem recognition^3^.

As minors in the U.S. mental healthcare system, youth already have less autonomy over their treatment choices and access to evidence-based care compared to adults. Further, youth with socially complex needs (SCN)^8^, who have endured adverse childhood experiences and/or faced multiple forms of marginalization due to socioeconomic status or minoritized identities, face the highest number of barriers to accessing mental healthcare^9^. In short, traditional mental health services have failed to reach all youth in need, especially those with SCN^1^.

## Digital mental health tools may increase access

The rise of DMH has poised itself as a means to address this mental healthcare access crisis^10^, even among individuals with SCN^11^. Indeed, DMH is a recently identified priority of the Biden administration, as technological devices are nearly ubiquitous in the U.S. and most youth own a smartphone by age 11^5^. DMH have also demonstrated efficacy in managing mental and physical conditions for pediatric and young adult samples in research settings^10^. Accordingly, DMH has often been touted as low-cost, scalable, easily accessible, and efficacious for mental healthcare delivery.

Despite research findings showing that DMH are efficacious in improving symptoms, this approach is not embraced as a solution by all. For instance, some argue that increases in technology use and time in online spaces have contributed to the youth mental health crisis. However, research has not coalesced around a robust conclusion to support this claim^12,13^. As such, quality (rather than quantity) of online experiences is likely the most salient factor for teen mental health. Given the positive findings in research settings, DMH may broadly be viewed as a positive technological experience for youth, as opposed to a detriment.

Other pressing issues with DMH are low reach and uptake. Teens in this generation, for example, were born during the advent of the smartphone, and are already highly engaged with online spaces, including social media. Youth are also more likely to seek emotional support through technology and online spaces^14^, implicating DMH accessibility and reach. Youth’s familiarity and comfort with digital spaces would, therefore, seemingly set up their generation to be the prime users of DMH, particularly in the current mental health crisis. Yet, DMH is rarely getting in the hands of youth^15^.

## A matter of justice: include youth in digital mental health

One of the main reasons why youth, particularly those with SCN, are not using evidence-based DMH is simple: these tools were not built for and with them. They have been systematically excluded from the design, research, and dissemination of DMH. Indeed, most publicly-available DMH have been tested on healthy adults^16^. Further, these adults are typically college-educated, healthy, White women–a demographic that differs from the unique experiences and needs of diverse youth. As youth are typically good assessors of authenticity, marketing DMH as *for* them without thoughtfully including these individuals in the development of DMH and ensuring cultural relevance may be quickly deemed inaccurate.

While there are recent examples of youth involvement in DMH design^17,18^, the level of involvement is typically isolated (e.g., a one-time session to provide feedback on a developed tool). Without sustained and collaborative engagement across the lifespan of a DMH innovation, questions are raised about this practice involving tokenism. Yet, these practices may be linked to youth being more complex than adult participants—they have specific digital preferences, developmental needs, constraints to their schedules, different levels of autonomy, varying literacy, and also require guardian consent to participate in DMH research. Especially, caregiver consent can represent a barrier to care and research participation due to issues ranging from fear of disclosing symptoms or marginalized identities to caregivers (e.g., sexual/gender minoritized identity)^19^ to having a non-English-speaking caregiver^20^. Thus, potential parental gatekeeping in DMH care can echo previously identified barriers to traditional mental healthcare that have been exacerbated for decades. As such, if DMH does not address injustices known to other forms of mental healthcare delivery, it is positioned to maintain or even exacerbate existing inequities.

Methods to increase representation and lived experience expertise in DMH already exist. Recent examples include the call by Figueroa and colleagues to apply a feminist intersectionality framework in digital health^21^ or the five key recommendations for the DMH field to “REACT” to reduce health disparities in racially and ethnically minoritized individuals by Friis-Healy and colleagues^22^. Both are examples of how addressing DMH justice requires better inclusion of specific patient populations in design and implementation, as well as increased mentorship, promotion, and representation of people with minoritized identities in DMH teams^10,21–25^.

Here, we make the case that similar principles apply to youth and are necessary to equitably address DMH implementation challenges that plague the literature. Specifically, to improve DMH uptake and engagement, particularly among minoritized youth, we must view youth as community partners in both DMH design and deployment. This perspective recognizes the necessity of youth’s contributions to ensure DMH may be usable in their everyday lives to support uptake^25^. To address reach, one avenue to address access barriers to mental healthcare for youth is making sure that care is consistently and affordably accessible digitally, where youth already are. Thus, this viewpoint emphasizes the value and recognition of their expertise by experience, as well as reframing their role in DMH design and deployment from one-time participants to integral community-based partners.

## Increasing uptake through youth partnership

Research and census data cannot compete with the insights youth know from their lived experiences^25^. As such, youth need to be included as partners to increase DMH uptake, with a focus on means to make tools engaging and appropriate to fit into the daily lived experiences of youth. First, aligned with CBPR recommendations^4^, this requires researchers and developers to power share and defer to the design priorities of youth. Second, this requires youth to be financially compensated for their participation in the design process, and to receive credit consistent with their contribution level (e.g., authorship). Third, purposeful recruitment for youth partners must ensure that youth with SCN are overrepresented in DMH design and deployment activities. Indeed, designing for diverse populations ensures for better generalizability^22,24^. To accomplish this goal, youth with SCN need to be able to see themselves represented in research and development team members and leadership, as well as potential design mock-ups of DMH^21–23,25^. Finally, caregivers should be included as collaborators, as they are key members of a youth’s family system and potential gatekeepers to technology access^26^. However, caregiver and youth data and input must be held confidentially, and dyad vs. single-person participation must be decided voluntarily by each individual. This list of methods to increase teen collaboration in DMH is far from exhaustive but does provide an initial roadmap to begin to address DMH inequities.

The issue of youth uptake with DMH needs to go beyond superficial DMH characteristics, such as appearance, to include youth’s lived experiences, as this crucial information will dictate if, when, and how they could use DMH for their mental health^27^. For example, some DMH could deplete a data plan if not connected to the Internet, yet not all youth have Wi-Fi at home. Differences in Internet access across communities vary drastically, with low-income, rural, and racially/ethnically minoritized households experiencing inequities^28^. These long-standing disparities have already set these youth behind in access to traditionally-delivered mental healthcare. Low socioeconomic status has been long implicated in the existence and exacerbation of poor health and relatedly highly-burdened experiences in accessing healthcare. As such, ignoring youth with SCN’s input perpetuates existing and recognized injustices.

## Increasing reach through youth partnership

While uptake is a critical domain to address, youth with SCN will be less likely to engage with DMH if they are never introduced to them in the first place. Systemic and individual-level barriers interfere with DMH reaching youth. This Comment focuses primarily on means to partner with youth to address individual-level barriers to DMH reach, but systemic and policy-level changes are critical to ensure justice in mental healthcare access.

Happily, efforts to increase reach may dovetail with formative and summative input from youth partners to increase DMH engagement. Indeed, methods to increase uptake make DMH more visible to peers; few things support youth buy-in as much as unsolicited recommendations from peers (i.e., word-of-mouth). Reach may also be influenced by the trusted individuals, professionals, and environments that youth encounter or seek out regularly. Particularly following remote learning and other pandemic experiences, youth with SCN have voiced an openness to teachers, physicians, and other trusted adults checking in about their mental health and making DMH recommendations^26^. Implementing evidence-based DMH screening and referral options into regularly attended and low-stigmatized settings, such as schools, libraries, community settings, primary care, and social media may therefore increase reach. Youth partners should regularly be encouraged to voice who their trusted sources and spaces are. As such, evidence-based DMH options and resources could be targeted toward the most relevant and impactful people and places for youth.

Importantly, when youth partners identify their trusted sources, DMH researchers and developers must strive to be on that list. Increasing reach via youth partnerships will only be successful if youth are treated as and believe themselves to be actual partners. Characteristics of successful partnership defined by CBPR include shared and trustworthy leadership, agility and adaptiveness in research activities and engagement in the community, and use of effective and ongoing communication^4^. Demonstrations of power sharing through this lens may involve leveraging platforms that youth use rather than requiring them to adopt use of a new digital service (i.e., deferring to the priorities of youth, even if that differs from researchers’ original agendas). Future research will be needed to refine how such methods equitably work in youth partnerships for DMH, including potentially navigating power dynamics and communication through remote connections.

## Conclusion

Injustices that have led to the inaccessibility of traditionally-delivered mental healthcare for youth are longstanding and compounded by recent endemics^1^. DMH stands as a potential means to address these systemic inequities. However, DMH can only accomplish this goal if reorganized to include youth, especially those with SCN^8,23^, as community partners. Without this purposeful and inclusive collaboration, DMH is likely to perpetuate injustices and continue to fall short in reach and uptake. The assumption that DMH tools will be scalable and engaging to youth because they are delivered on smartphones is insufficient and unfounded. At worst, these assumptions can be harmful if “go digital” becomes the default strategy to circumvent the inequities of face-to-face interventions--without appreciating the complexities of implementing such tools with youth. Indeed, barriers to DMH actually being used and helpful for youth cannot be overcome without working to make youth—particularly those from minoritized backgrounds^22^—feel welcome and wanted at the digital table.
